# Pan-*Parastagonospora* Comparative Genome Analysis—Effector Prediction and Genome Evolution

**DOI:** 10.1093/gbe/evy192

**Published:** 2018-09-04

**Authors:** Robert A Syme, Kar-Chun Tan, Kasia Rybak, Timothy L Friesen, Bruce A McDonald, Richard P Oliver, James K Hane

**Affiliations:** 1 Centre for Crop & Disease Management, School of Molecular & Life Sciences, Curtin University, Bentley, Western Australia, Australia; 2 Cereal Crops Research Unit, USDA-ARS Red River Valley Agricultural Research Center, Fargo, North Dakota; 3 Plant Pathology Group, Institute of Integrative Biology, Swiss Federal Institute of Technology (ETH), Zurich, Switzerland; 4 Curtin Institute for Computation, Curtin University, Bentley, Western Australia, Australia

**Keywords:** *Parastagonospora nodorum*, Pan-genome, plant pathogen, crop disease, host–microbe interactions

## Abstract

We report a fungal pan-genome study involving *Parastagonospora* spp., including 21 isolates of the wheat (*Triticum aestivum*) pathogen *P*arastagonospora *nodorum*, 10 of the grass-infecting *Parastagonospora avenae*, and 2 of a closely related undefined sister species. We observed substantial variation in the distribution of polymorphisms across the pan-genome, including repeat-induced point mutations, diversifying selection and gene gains and losses. We also discovered chromosome-scale inter and intraspecific presence/absence variation of some sequences, suggesting the occurrence of one or more accessory chromosomes or regions that may play a role in host–pathogen interactions. The presence of known pathogenicity effector loci *SnToxA, SnTox1*, and *SnTox3* varied substantially among isolates. Three *P. nodorum* isolates lacked functional versions for all three loci, whereas three *P*. *avenae* isolates carried one or both of the *SnTox1* and *SnTox3* genes, indicating previously unrecognized potential for discovering additional effectors in the *P. nodorum*-wheat pathosystem. We utilized the pan-genomic comparative analysis to improve the prediction of pathogenicity effector candidates, recovering the three confirmed effectors among our top-ranked candidates. We propose applying this pan-genomic approach to identify the effector repertoire involved in other host–microbe interactions involving necrotrophic pathogens in the Pezizomycotina.

## Introduction

### The *Parastagonospora* Pathosystems

*Parastagonospora* (teleomorph: *Phaeosphaeria*) *nodorum* (Berk.) is an economically important necrotrophic fungal pathogen that causes septoria nodorum blotch (SNB) in wheat (*Triticum aestivum*) (Solomon et al. 2006) and is also a model organism for the fungal order Pleosporales and for necrotrophic phytopathogenicity (Oliver and Solomon 2010; Tan et al. 2010; Oliver et al. 2012). Significant experimental resources are available for *P. nodorum*, including a high-quality genome assembly of the reference strain SN15 (Hane et al. 2007; Syme et al. 2016), microarray analyses of gene expression (Ipcho et al. 2012), proteomics and proteogenomics, metabolomic profiling, and comparative genomics versus two contrasting *P. nodorum* strains: Sn4 and Sn79-1087 (Syme et al. 2013; Richards et al. 2018). SN15 and Sn4 are highly aggressive isolates on wheat, whereas Sn79-1087 (hereafter referred to as Sn79) was isolated from the wild grass *Agropyron,* is avirulent on wheat, and serves as a useful negative control for comparative genomics in a disease context (Friesen et al. 2006; Richards et al. 2018).

In this study we resequenced the genomes of 18 new strains of *P. nodorum* as well as 10 strains representing closely related species within the *Parastagonospora* genus (table 1). *Parastagonospora avenae* is a species associated with SNB-like symptoms in various Poaceae spp. (Quaedvlieg et al. 2013). *P. avenae* was further subdivided into two *formae speciales*. *P. avenae* f. sp. *avenaria* (*Paa*) infects oats (*Avena* spp.) and *P. avenae* f. sp. *triticea* (*Pat*) infects wheat and some other grasses (Cunfer 2000; Ueng et al. 2003; Malkus et al. 2005). Restriction fragment length polymorphism patterns were used to further differentiate *Pat* strains into distinct subgroups named *Pat1*-*Pat6* (Ueng and Chen 1994; Ueng et al. 1995; Ueng et al. 1998). Two isolates of a sister species, provisionally named *P2* (*Phaeosphaeria 2*) were collected from Iranian wheat fields. The placement of *P2* is not yet clear, but sequences of the β-tubulin and β-xylosidase loci were similar to *P. nodorum* and *P. avenae* (McDonald et al. 2012).

**Table 1:** Summary of Source Material and Resequencing Data for Strains of (A) *P. nodorum*, (B) *P. avenae*, and (C) the *P2* clade

Isolate ID	Source	Collection Year	Total Length of Error-corrected Reads (Mb)	Estimated Genome Coverage (X)	Isolation Source	Contributor
**(A) *P. nodorum* strains**						
B2.1b	Iran	2005	686.1	9.2	*T. aestivum*	Mohammad Razavi, Iranian Research Institute of Plant Protection
C1.2a			886.1	11.9		
IR10_9.1a		2010	528.4	7.1		
FIN-2	Finland	unknown	1,998.7	26.9		Andrea Ficke, Nibio, As, Norway
SWE-3	Sweden		1,517.8	20.4		
Sn Cp2052			1,031.9	13.9		Hans Jorgensen, University of Copenhagen
BRSn9870	Brazil		3,316.9	44.6		Flavio Santana, EMBRAPA
Sn99CH 1A7a	Switzerland	1999	3,888.2	52.3		Bruce McDonald, ETH Zurich
SnChi01 40a	China	2001	2,796.4	37.6		R. Wu, Louyuan, Fujian Province
SnSA95.103	South Africa	1994	1,019	13.7		Pedro Crous, University of Stellenbosch, South Africa
AR1-1	Arkansas, USA	2011	1,986.2	26.7		Christina Cowger, USDA-ARS
GA9-1	Georgia, USA		4,296.5	57.8		
MD4-1	Maryland, USA	2012	4,325.8	58.2		
VA 5-2	Virginia, USA		2,288.3	30.8		
OH03 Sn-1501	Ohio, USA	2003	2,229.8	30		Pat Lipps, Ohio State University
SNOV92X D1.3	Texas, USA	1992	2,524.6	34		Bruce McDonald, Texas A&M University
SnOre11-1	Oregon, USA	2011	4,763.4	64.1		–
WAC8410	Australia	2010	6,032.4	81.2		Department of Agriculture and Food, Western Australia
**(B) *P. avenae* strains**						
**(i) *P. avenae* f. sp. *triticea 1***						
IR10_5.2b	Iran	2010	850.5	11.4	*T. aestivum*	Mohammad Razavi, Iranian Research Institute of Plant Protection
Hartney99	Canada	2005	1,132.5	15.2	*T. aestivum* (spring wheat)	–
Jansen #4_55			480.1	6.5		–
**(ii) *P. avenae* f. sp. *triticea 4***						
SN11IR_2_1.1	Iran	2011	1,415.3	19	*Dactylis glomerata*	Mohammad Razavi, Iranian Research Institute of Plant Protection
**(iii) *P. avenae* f. sp. *triticea 5***						
82-4841	North Dakota, USA	1982	1,207.7	16.2	Unknown grass	Joe Krupinsky, USDA-ARS
83-6011-2		1983	1,136.3	15.3	*Bromus* (Brome grass)	
***iv) P. avenae* f. sp. *triticea 6***						
SN11IR_6_1.1	Iran	2011	2,150.2	28.9	*Agropyron tauri*	Mohammad Razavi, Iranian Research Institute of Plant Protection
SN11IR_7_2.3	Iran	2011	1,039.4	14	*Dactylis glomerata*	
**(v) *P. avenae* f. sp. *Avenaria***						
Mt. Baker	Washington, USA	2009	585.8	7.9	*Avena sativa*	–
s258	Netherlands	2005	1,163.2	15.6		–
**(C) *P2* strains (**McDonald et al. 2012**)**						
A1 3.1a	Iran	2005	1,813.1	24.4	*T. aestivum*	Mohammad Razavi, Iranian Research Institute of Plant Protection
H6.2b		2005	3,030	40.8		

Many of the recent studies of *Parastagonospora* spp. are oriented around an important class of genes encoding effector molecules that interact with the host to determine the outcome of specific host–pathogen interactions (Vleeshouwers and Oliver 2014). A class of effectors called necrotrophic effectors (NEs) interacts with host dominant susceptibility genes to form an inverse gene-for-gene interaction (Oliver and Solomon 2010; Tan et al. 2010). A compatible interaction results in plant cell death and subsequently, infection. *Parastagonospora* spp. were already shown to produce several NEs (Friesen et al. 2007), which are thought to maximize the likelihood of interacting with a corresponding susceptibility protein in the host. So far, three well characterized NE genes have been identified in *P. nodorum*: *SnToxA* (*SNOG_16571*) (Friesen et al. 2006), *SnTox1* (*SNOG_20078*) (Liu et al. 2004; Liu et al. 2012), and *SnTox3* (*SNOG_08981*) (Friesen et al. 2008; Liu et al. 2009). These three effectors are already used in breeding programs to accelerate development of disease-resistant cereal cultivars (Vleeshouwers and Oliver 2014), but several NE loci have not yet been identified to the gene level, including *SnTox2* (Friesen et al. 2007), *SnTox4* (Abeysekara et al. 2009), *SnTox5* (Friesen et al. 2012), *SnTox6* (Gao et al. 2015), and *SnTox7* (Shi et al. 2015). Although continued laboratory testing may yield new effectors, further advances in genome sequencing technologies and bioinformatic methods may also improve effector discovery (Jones et al. 2018). This study provides enhancements to the *P. nodorum* SN15 reference strain assembly and its gene annotations, but also explores features of the *Parastagonospora* fungal genomes that are relevant for effector discovery, including repeat-induced point mutation (RIP), presence–absence variation (PAV), and diversifying selection.

RIP is a fungal-specific form of mutation that targets repetitive sequences and introduces cytosine to thymine (C → T) transitions, or the reverse complement G → A. In the filamentous Ascomycota (syn. Pezizomycotina) where RIP is commonly observed (Testa et al. 2016), there is a strong bias for mutations at cytosine bases adjacent to adenine (CpA → TpA). RIP provides a mechanism of genome defence against transposon invasion, by disabling transposable elements through introduction of premature stop codons into their open reading frames and/or through silencing of the RIP-mutated sequence through further DNA methylation (Galagan and Selker 2004; Hane and Oliver 2008; Clutterbuck 2011; Hane 2015; Hane et al. 2015). RIP has also been linked to mutation of avirulence effector genes *AvrLm1*, *AvrLm6*, and *AvrLm4-7* in *Leptosphaeria maculans* through leakage into nonrepetitive regions flanking repeats (Fudal et al. 2009; Van de Wouw et al. 2010). Fungal genes most likely to be affected by RIP leakage can be identified by annotation of AT-rich regions with OcculterCut (Testa et al. 2016).

**Fig. 1.**
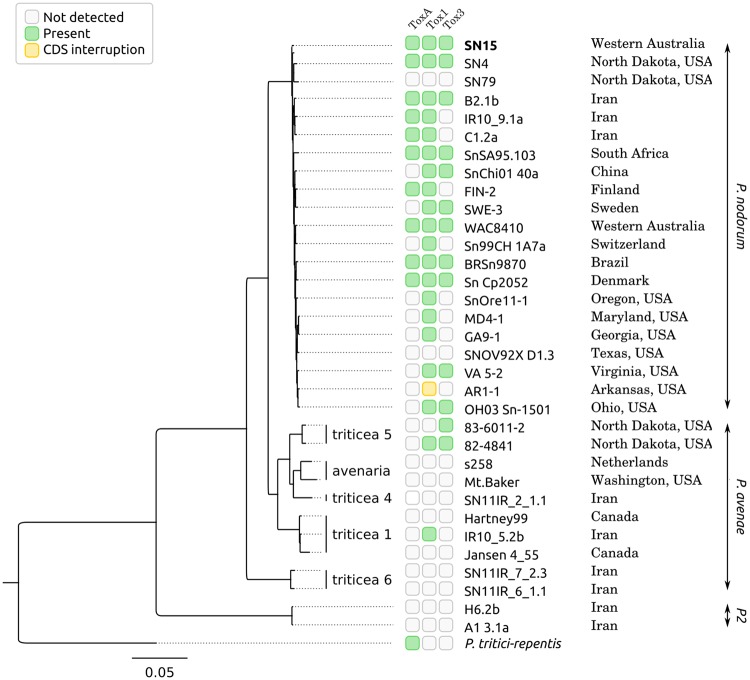
—A phylogeny of the *P. nodorum*, *P. avenae*, and *P2* strains used in this study and the presence or absence of known effector loci: *ToxA*, *Tox1*, and *Tox3*. Green boxes indicate the presence of a known effector locus in that strain and yellow box indicates the presence of a psuedogenized version.

Recent resequencing studies have repeatedly shown that PAV in gene content is common in Fungi (McDonald et al. 2013; Gao et al. 2015; Golicz et al. 2015). PAV patterns have been observed at both the gene cluster level (Plissonneau et al. 2016) and at whole (or partial) chromosome level (Ma et al. 2010; Goodwin et al. 2011), and may indicate variable effector loci when applied across multiple isolates of a single species with a range of virulence phenotypes. Sectional absences of small groups of genes were previously reported between the reference strain SN15 and other strains of *P. nodorum* (Syme et al. 2013). PAVs related to known effectors vary in length, with *SnTox1* and *SnTox3* absent from the wheat-avirulent strain Sn79 in small 2 and 4 kb stretches, respectively, whereas *SnToxA* is part of a much larger 72 kb absence in Sn79 (Syme et al. 2013). The PAV pattern of known effectors also varies in field populations of *Parastagonospora* spp. (fig. 1), which may indicate multiple, independent horizontal gene transfer (HGT) events (McDonald et al. 2013). Notably, there does not appear to be a significant fitness penalty incurred by the pathogen harboring an effector when growing on a host that lacks the corresponding sensitivity gene (McDonald et al. 2013). Genes within repeat-rich regions may be more prone to loss due to mesosyntenic recombination (Hane et al. 2011) or breakage fusion bridge cycles (Bertazzoni et al. 2018).

The genomes of some pathogenic species are compartmentalized into regions with “two speeds” of evolution: “core” gene content—which tends to be well-conserved among strains and under purifying selection, and “accessory” gene content—which typically is repeat-rich and exhibits both PAV and evidence for diversifying selection. For some species, such as *L. maculans*, these variable regions are interspersed throughout the genome. In other species, accessory and core gene contents are divided among separate chromosomes (Bertazzoni et al. 2018). Fungal accessory (syn. supernumerary or dispensable) chromosomes have been observed to be gene sparse, repeat-rich, and exhibit higher rates of mutation, positive selection and pathogenicity-associated loci relative to the “core” chromosomes (Bertazzoni et al. 2018). The *P. nodorum* isolates in this study may also contain accessory sequences, as wheat- and barley-infecting isolates have been previously reported to possess an accessory chromosome of approximately 400 kb (Zolan 1995). Furthermore, *P. nodorum* SN15 scaffold_46 was previously predicted to be dispensable based on sharing common characteristics with *Z. tritici* accessory chromosomes, including low GC content, high repeat content, low gene density and a low percentage of predicted genes with conserved functional domains (Ohm et al. 2012).

Recent advances in understanding fungal genome evolution coupled with improved criteria for identifying candidate effector genes provide new opportunities to understand the role of intraspecies genome diversity in the context of pathogenicity. Here, we compare 19 isolates of *P. nodorum*, 10 isolates of *P. avenae*, and 2 isolates of the closely related sister group P2.

## Materials and Methods

### Strain Sampling, DNA Extraction and Sequencing

Illumina paired-end libraries were constructed for each haploid strain. *P. nodorum* WAC8410 was sequenced from a TruSeq 500 bp library on an Illumina 2000 to produce 150 bp paired-end reads. All other strains were sequenced from 300 bp NexteraXT libraries on an Illumina 2500 multiplexed over two lanes.

### Reference-Alignment and *de novo* Assembly of Strain Sequences

Raw Illumina reads were filtered for PCR duplicates, then trimmed using cutadapt v1.7.1 (Martin 2011), removing the adapter sequences, bases with quality scores less than 25, and any reads shorter than 50 bp. *P. nodorum* strain SN15 (Hane et al. 2007) was used as the reference against which each of the newly sequenced strains was compared. The annotated gene set of SN15 has been improved with transcriptomic and proteomic data, and manual curation of gene models (Syme et al. 2016). Trimmed reads were aligned to the reference genome using bowtie2 (Langmead and Salzberg 2012) (“very-sensitive”). Trimmed reads were also assembled *de novo* with SPAdes v.3.5.0 (Bankevich et al. 2012) (kmers 21, 33, 55, and 77) using mismatch and short indel correction via BWA mapping (Li 2013). Assembly quality was assessed with QUAST v2.3 (Gurevich et al. 2013). Genome assemblies were aligned to the reference with MUMmer v3.0 (nucmer) (Kurtz et al. 2004) and repetitive matches were filtered with delta-filter (–r and –q). SNP and indel variants from genome alignments were determined with MUMmer (show-snps). PAV profiles were generated by calculating the coverage of each of the SN15 reference scaffolds by nucmer matches to each isolate assembly using the genomecov function of BEDtools (Quinlan and Hall 2010). Homologs of known effectors were extracted from orthologous clusters. Each effector absence was manually confirmed by blasting the reference effector against the new strain’s genome assembly.

### Genome Annotation

Manually curated annotations from the reference genome SN15 (Syme et al. 2016) were used to train CodingQuarry (Testa et al. 2015) and these parameters were applied to each of the alternate strains. A database of repeats was generated using RepeatModeler v1.0.8 (Smit and Hubley 2010) augmented with full copies of the known *P. nodorum* repeats Molly (AJ488502.1), Pixie (AJ488503.1), and Elsa (AJ277966.1). The RepeatModeler repeats were combined with known “DeRIPped” (predicted pre-RIP consensus) (Hane and Oliver 2010) and repbase (Jurka et al. 2005) repeats using RepeatMasker v4.0.5 (Smit et al. 2015). A final annotation set for each alternate strain (supplementary file S1, Supplementary Material online) was generated using Augustus v3.3 (Stanke et al. 2006) using annotation hints provided CodingQuarry annotations, exonerate v2.2.0 protein matches (Slater and Birney 2005) to the SN15 predicted proteome and from RepeatMasker matches described above. Secretion signals were detected using SignalP v4.1 (Bendtsen et al. 2004) and transmembrane domains by TMHMM v2.0 (Krogh et al. 2001). Secondary metabolite clusters were predicted in nonreference strains by antismash 2.1.1 (Medema et al. 2011). Orthofinder v2.2.3 (Emms and Kelly 2015) using Diamond v0.9.21 (Buchfink et al. 2015) for distance estimation was used to predict clusters of orthologous proteins and to generate a phylogeny of the isolates used in this study, using the closely related species *Pyrenophora tritici-repentis* as an outgroup (Moolhuijzen et al. 2018). In the case of known effector loci *SnToxA*, *SnTox1* and *SnTox3,* their ortholog cluster membership was manually inspected by tblastn (Altschul et al. 1990) (e-value cutoff 1 × 10^−5^) queries of their translated sequences versus each of the alternate strain *de novo* assemblies.

### Identification of Genes under Diversifying Selection

Coding sequences from all strains that were orthologous to protein-coding genes from the reference strain were extracted, translated, and aligned using ClustalW (Larkin et al. 2007). Protein truncations due to incorrect annotation in a novel strain can limit detection of diversifying selection, so only codons present in all strains were considered. Short proteins with lengths more than 1 standard deviation from the mean were excluded from these alignments to reduce the frequency of false-positive results.

Codon alignments were generated with PAL2NAL v14 (Suyama et al. 2006). The M1a and M2a site models were applied to codon-aligned transcripts of orthologous proteins to generate a maximum likelihood (ML) estimation of *ω*. The H_0_ model (PAML model M1a) confines codon membership to one of two classes where *ω* < 1 (purifying selection) or *ω* = 1 (neutral/drift). The H_1_ model (PAML model M2a) extends H_0_ to allow codon membership to a third possible class where *ω* > 1 (diversifying selection). Loci with sites under diversifying selection were identified where the *χ*^2^-distributed likelihood ratio of the two models exceeded the 1% significance level (2 degrees of freedom). Local patterns of selection were identified by stepping a 100 kbp window over the reference assembly in 1 kbp increments and counting the number of transcripts under diversifying selection as a percentage of the total number of transcripts in each window.

### Prediction of Effector Candidates

For each reference transcript sequence, a number of tests were applied and a cumulative score was generated based on the number of passed tests. Protein scores were increased if they had a molecular mass less than 30 kDa, had a cysteine percentage greater than 4%, were longer than 200 bp, did not have tblastn hits to the wheat-avirulent Sn79 genome assembly (e-value ≤ 1 × 10^−30^), were in regions of low gene density (no genes predicted within the 2 kb region(s) up/downstream), were predicted to be secreted by SignalP v4.1 (Bendtsen et al. 2004), were predicted to be under positive diversifying selection pressure as described above, were not part of the core proteome, or were not predicted to encode a transmembrane domain by TMHMM (Krogh et al. 2001), were less than 5 kb from the nearest AT-rich region as predicted by OcculterCut, or had an EffectorP score greater than 0.9.

## Results

### Pan-Genome Resources Generated for Multiple Isolates of *P. nodorum*, *P. avenae* and *P2*

The *Parastagonospora* strains originated from a broad geographical range with a focus on the Fertile Crescent and the United States (table 1 and fig. 1) and included three previously sequenced *P. nodorum* strains (Syme et al. 2016; Richards et al. 2018), 18 newly sequenced *P. nodorum* strains, 10 newly sequenced *P. avenae* strains, and 2 newly sequenced strains of the closely related *P2* group (McDonald et al. 2012) (BioProject: PRJNA476481). The estimated genome coverage for each strain, relative to the SN15 reference assembly, ranged between 7–64X for strains sequenced using the NexteraXT libraries and 81X for the WAC8410 isolate sequenced using a separate TruSeq library (table 1). Total genome assembly length ranged from 33.5 to 49.9 Mb (table 2).

**Table 2:** Summary of Genome Assembly Metrics for Resequenced Strains of (A) *P. nodorum*, (B) *P. avenae*, and (C) the P2 clade

Isolate ID	№ Scaffolds	Largest Scaffold (kb)	Total Length (Mb)	N50 (kb)	Whole SN15 Gene Count by QUAST	Partial SN15 Gene Count by QUAST
**(A) *P. nodorum* strains**						
B2.1b	2,906	386.4	37.38	60.3	12,628	784
C1.2a	1,557	325.3	37.43	80.4	12,751	649
IR10_9.1a	3,673	140.6	37.28	20.7	11,442	1,920
FIN-2	1,381	451.6	38.41	117.9	12,952	479
SWE-3	1,714	335.5	37.85	58.8	12,734	727
Sn Cp2052	3,026	190.2	37.32	38.2	12,310	1,148
BRSn9870	4,911	309.9	41.23	52.4	12,342	1,148
Sn99CH 1A7a	853	521	37.9	180.7	13,062	361
SnChi01 40a	779	1,268.10	37.88	206.3	13,118	332
SnSA95.103	11,772	48	49.94	6.5	9,545	3,882
AR1-1	882	748.5	36.61	135.6	12,999	412
GA9-1	664	938.6	36.53	215.3	13,074	356
MD4-1	701	599.9	36.53	191	13,045	384
VA 5-2	1,115	485.6	36.48	89.1	12,787	606
OH03 Sn-1501	1,281	349.9	37.1	85	12,844	577
SNOV92X D1.3	785	524.1	36.66	145.6	13,009	442
SnOre11-1	748	875.4	37.42	249.6	13,103	352
WAC8410	384	1,060.50	40.27	316.8	13,223	277
**(B) *P. avenae* strains**						
**(i) *P. avenae* f. sp. *triticea* 1**						
IR10_5.2b	1,681	267	35.51	57.3	18	14
Hartney99	3,381	124.3	36.58	27.6	47	20
Jansen 4_55	10,109	83.1	32.06	4.3	20	166
**(ii) *P. avenae* f. sp. *triticea* 4**						
SN11IR_2_1.1	5,762	168.2	41.54	38.6	12	15
**(iii) *P. avenae* f. sp. *triticea* 5**						
82-4841	2,444	218.3	38.53	50.5	21	13
83-6011-2	2,367	193.8	37.52	43.9	21	13
**(iv) *P. avenae* f. sp. *triticea* 6**						
SN11IR_6_1.1	1,174	737.1	33.51	102.8	3	7
SN11IR_7_2.3	2,215	244.5	33.6	44.8	6	12
**(v) *P. avenae* f. sp. *Avenaria***						
Mt. Baker	8,309	38.5	34.14	6.2	15	81
s258	4,090	183.1	39.49	32.8	16	21
**(C) *P2* strains**						
H6.2b	1,613	411.9	38.68	74.6	2	6
A1 3.1a	1,764	419.9	39.05	68.3	3	6

Note.—*P. avenae* and *P2* strains show a low number of *P. nodorum* gene matches from QUAST due to dissimilarities in the coding sequence relative to the *P. nodorum* SN15 reference.

### *SnToxA, SnTox1*, *and SnTox3* Effector Loci Vary in Their Distribution Across Both Taxonomic Groups and Geographic Locations

**Fig. 2.**
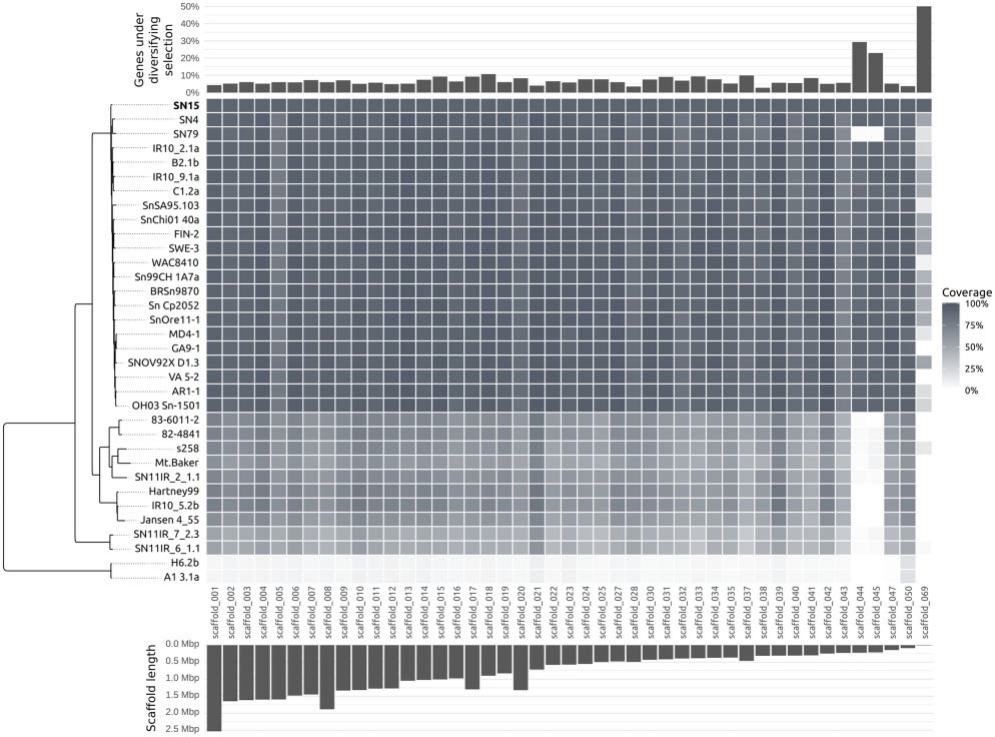
—Coverage of reference *P. nodorum* scaffolds by nucmer matches from the alternate strains. Scaffolds 44 and 45 (putatively dispensable) and Scaffold 51 (contains *ToxA*) are absent from the wheat-avirulent *P. nodorum* strain SN79, and all *P. avenae* and *P2* strains.

The ortholog cluster containing the *SnToxA* effector gene was found in 10 *P. nodorum* strains (48%) but no *P. avenae* strains. Intact *SnTox1* gene sequences were detected in 18 *P. nodorum* strains (86%) and 2 *P. avenae* strains (17%). The *SnTox3* gene was detected in 11 *P. nodorum* strains (52%) and the 2 *P. avenae* Pat5 strains (figs. 1 and 2).

### Presence/Absence Patterns Across the *Parastagonospora* Pan-Genome Indicate Putative Lineage-Specific Accessory Sequences

There were differing outcomes for sectional absence based on the read-mapping methods employed in previous studies (Croll and McDonald 2012; Croll et al. 2013; McDonald et al. 2015; Syme et al. 2016) compared with *de novo* assembly and subsequent alignment via nucmer used here. For comparisons between *P. nodorum* strains and the SN15 reference, the read-mapping method was able to genotype 90.3% of the genome. In contrast, the nucmer method found fewer sectional absences and was able to identify variants over 93.5% of the genome. In more distant comparisons of *P. avenae* strains to the SN15 reference, read-mapping was able to genotype only 16.2% of the genome and nucmer was able to find variants over 61.9% of the genome.

**Fig. 3.**
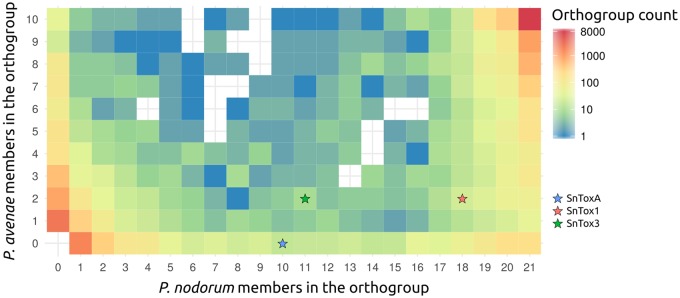
—Presence and absence of protein orthologs between *P. nodorum* and *P. avenae* strains. The number of *P. nodorum* strains that have contributed a protein to the cluster determines the *x*-axis location and the number of *P. avenae* strains that have contributed a protein to the cluster determines the *y*-axis location. Core conserved genes with members from all strains are at the top-right and strain-specific genes are at the bottom-left. About 10,798 (53.8%) of clusters are missing from at most 2 strains. About 6,229 clusters (29.0%) are present in at most 3 strains. The three known *P. nodorum* effectors (red, blue, and green stars) are present in only some of the isolates.

Of the 51 *P. nodorum* SN15 reference scaffolds that contained genes, most had consistent nucmer match coverage by sequences of the resequenced strains. Scaffolds 44, 45, 51, 57, 69, 97, 99, and 101 had very low coverage by alignment of *P. avenae* and *P2* isolates (fig. 3). These scaffolds are composed largely of repeats, except scaffolds 44 and 45 which respectively encode 75 and 61 genes and contain only 1.9% and 2.4% repetitive sequence. Scaffolds 44, 45 also had a high percentage of genes under diversifying selection (29.3% and 23.0%). The wheat-avirulent *P. nodorum* strain SN79 also covered scaffolds 44 and 45 over 6.7% and 5.0% of their lengths, respectively, compared with 94.4% and 96.3% coverage by the other *P. nodorum* strains.

### Patterns of Strain-Specificity and Positive Selection Across the Pan-Genome Refines Effector Candidate Predictions

**Fig. 4.**
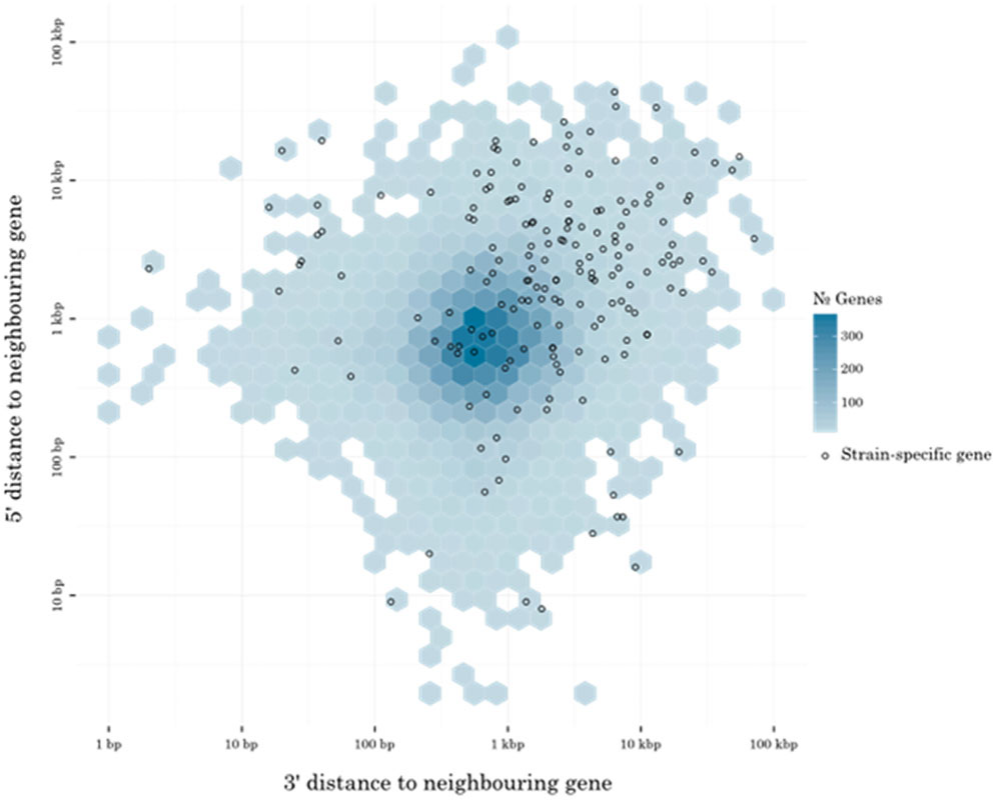
—Intergenic distances at the 3′ and 5′ ends for all genes of *P. nodorum* WAC8410. Intergenic distances at 3′ and 5′ for all WAC8410 genes. Hexagonal cells are colored to indicate the number of genes that have flanking intergenic distances that place it within the cell’s bounds. Strain-specific genes (highlighted as hollow circles) generally have higher distances to the neighboring gene, placing them in the top-right quadrant. The mean intergenic flank distance is 6,558 bp for strain specific genes and 1,162 bp for all other genes.

**Fig. 5.**
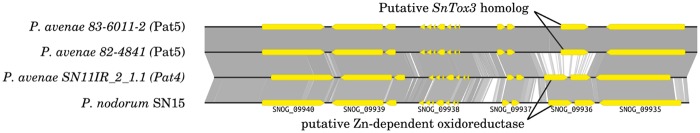
—Alignment of genes surrounding the *P. avenae* Pat5 *Tox3* homolog with the corresponding loci from the *P. nodorum* SN15 reference and a representative *P. avenae* Pat4 region. The *Tox3* gene is absent from the Pat4 genome completely, and present in the SN15, but on a different scaffold to the one shown here.

The distribution of conserved proteins between the *P. avenae* and *P. nodorum* strains shows most proteins are either strain-specific or are well conserved, but known effectors are present in the sparsely populated middle-ground (fig. 4). Of the 21,470 orthologous protein groups, 6,229 (29.0%) were observed in fewer than four strains, 12,473 (58.1%) were missing from at most one strain, and 6,620 (30.8%) were between the two extremes (table 3 and fig. 4). *P. nodorum* strain WAC8410 has 184 strain-specific loci (supplementary file S1 and table S2, Supplementary Material online). This strain was sequenced at higher coverage (table 1) and is a more complete assembly (table 2) than the other strains, enabling a more detailed study of its strain-specific loci. The mean intergenic flanking distance for all WAC8410 genes is 1,162 bp. The mean intergenic flanking distance for the strain-specific subset of WAC8410 genes is 6,558 bp (fig. 5). WAC8410 scaffolds 50 and 55 are rich in strain-specific genes, with 18 of 20 and 11 of 23, respectively. At least 8 of the 18 strain-specific genes on scaffold 55 appear to be pseudogenes with high rates of mutation and premature stop codons. Included in the scaffold 55 strain-specific set are a pseudogene which shows similarity to the *P. nodorum* PnPf2 transcription factor (Rybak et al. 2017), an indole-diterpene biosynthesis protein, and a pseudogene which shows similarity to *SNOG_08983* which, in SN15, is notably near to the *SnTox3* locus. Effector candidates were predicted from the SN15 reference protein set by using a series of criteria based on the expected characteristics of NEs (table 4). Scores were calculated for 13,949 SN15 proteins, of which: 616 were cysteine-rich; 451 were associated with AT-rich regions; 798 were absent from Sn79; 2,414 resided in regions of low gene density; 1,475 were predicted as secreted; and 945 were under positive selection. Effector candidate scores are presented in full in supplementary table S3, Supplementary Material online, and 63 candidates with scores of 5 and above are presented in table 5. Known effector genes *SnToxA*, *SnTox1*, and *SnTox3* all scored highly using this system (table 5). Of the *P. avenae* isolates sequenced, only the wheat-avirulent Pat5 genomes (McDonald et al. 2012) contained putative *SnTox3* orthologs (fig. 1). Interestingly, the genomic context of this gene in *P. avenae* is different compared with the *SnTox3* positive *P. nodorum* strains. The putative *SnTox3* ortholog in *P. avenae Pat5* is in the position of a Zn-dependent oxidoreductase in *P. nodorum* and the other *P. avenae* genomes (fig. 5).

**Table 3:** Summary of Protein Conservation Across the *P. nodorum* and *P. avenae* Strains

Reference Protein Set	13,836 proteins
*Core Phaeosphaeria protein set*	
Missing from 0 strains	8,660 clusters
Missing from at most 1 strain	9,921 clusters
Missing from at most 2 strains	10,798 clusters
*Core P. nodorum protein set*	
Missing from 0 *P. nodorum* strains	11,366 clusters (11,821 SN15 proteins)
Missing from at most 1 *P. nodorum* strain	12,473 clusters (12,877 SN15 proteins)
Missing from at most 2 *P. nodorum* strains	13,049 clusters (13,139 SN15 proteins)
*Strain-specific protein set*	
Observed in only 1 strain	3,995 clusters (108 SN15 proteins)
Observed in at most 2 strains	5,438 clusters (240 SN15 proteins)
Observed in at most 3 strains	6,229 clusters (291 SN15 proteins)
*Dispensable protein set (effector-containing set)*	
Observed in between 4 and 30 strains (inclusive)	6,620 clusters (321 SN15 proteins)
*P. nodorum-specific proteins*	
Present in all nodorum, absent from all *P. avenae*	216
*P. avenae-specific proteins*	
Present in all *P. avenae*, absent from all *P. nodorum*	44

Note.—About 8660 protein clusters are observed in all strains. There are 204 protein clusters containing members of only one species. The set of “dispensable” proteins is defined here as proteins that are not species-specific (observed in fewer than four isolates) and not well conserved (missing in fewer than three isolates). This “dispensable” set of 2192 proteins contains 213 SN15 proteins, including all of the known effectors.

**Table 4:** Counts of the Numbers of SN15 Reference Proteins That Match Each Effector Prediction Criteria

Criteria	№ proteins
**Positive scores**	
Small—less than 30 kDa	4,362
Cysteine-rich—encodes an amino acid with >4% cysteine residues	616
Near repeats—less than 5 kb from repetitive sequence	3,417
Absent from SN79—no blast hits to the avirulent strain	798
Low gene density—encoded in a region with large intergenic space	2,414
Secreted—includes a signal peptide	1,475
Under positive selection	945
EffectorP (subset of secreted)	288
OcculterCut proximity to GC-AT border	451
**Negative scores**	
Core Set—missing in at most one strain	10,294
Strain specific—only found in SN15	108
Membrane bound—not predicted to encode a transmembrane domain	2,381

Note.—Each predicted protein is assessed against each of these criteria and assigned a total score calculated as the sum of the criteria scores (table 5).

**Table 5:** Top Effector Candidates with Scores ≥5

Locus	Predicted Secreted	Absent in SN79	<1 Gene/2 kb	≤30 kDa	Positive Selection	≥4% Cys	AT-Rich Regions	Effector P Score ≥0.9	Candidate Score ≥5
***SNOG_20078 (SnTox1)***	✓	✓	✓	✓	✓	✓	–	✓	7
*SNOG_01124*	✓	–	✓	✓	✓	✓	✓	✓	7
*SNOG_11452*	✓	✓	✓	✓	–	✓	✓	✓	7
*SNOG_11453*	✓	✓	✓	✓	–	✓	✓	✓	7
*SNOG_30077*	✓	✓	✓	✓	–	✓	✓	✓	7
*SNOG_30343*	✓	–	✓	✓	–	✓	✓	–	7
*SNOG_30466*	✓	✓	✓	✓	✓	✓	–	✓	7
*SNOG_30828*	✓	✓	✓	✓	–	✓	✓	✓	7
***SNOG_16571 (SnToxA)***	✓	✓	✓	✓	–	–	✓	✓	6
***SNOG_08981 (SnTox3)***	✓	✓	✓	✓	–	–	✓	✓	6
*SNOG_01097*	✓	–	✓	✓	✓	–	✓	✓	6
*SNOG_07039*	✓	✓	✓	✓	–	✓	–	✓	6
*SNOG_12811*	✓	–	✓	✓	✓	✓	–	✓	6
*SNOG_16520*	✓	✓	–	✓	✓	–	✓	✓	6
*SNOG_20100*	✓	✓	✓	✓	–	✓	–	✓	6
*SNOG_30802*	✓	✓	–	✓	–	✓	✓	✓	6
*SNOG_30888*	✓	✓	✓	✓	–	✓	–	✓	6
*SNOG_00726*	✓	–	✓	✓	✓	–	–	✓	5
*SNOG_03114*	✓	–	–	✓	–	✓	✓	✓	5
*SNOG_04279*	✓	✓	✓	✓	–	–	–	✓	5
*SNOG_04353*	✓	–	✓	✓	–	✓	–	✓	5
*SNOG_05030*	✓	✓	✓	✓	–	✓	–	–	5
*SNOG_06202*	✓	–	✓	✓	–	✓	–	✓	5
*SNOG_06459*	✓	✓	–	✓	–	✓	–	✓	5
*SNOG_07292*	✓	–	✓	✓	–	✓	–	✓	5
*SNOG_08206*	✓	–	✓	✓	–	✓	–	✓	5
*SNOG_08469*	–	–	✓	✓	–	–	✓	–	5
*SNOG_08606*	✓	✓	–	✓	–	✓	–	✓	5
*SNOG_09147*	✓	–	✓	✓	–	✓	–	✓	5
*SNOG_09446*	–	–	✓	✓	✓	✓	✓	–	5
*SNOG_09672*	✓	✓	–	✓	–	✓	–	✓	5
*SNOG_09738*	✓	✓	–	✓	–	✓	–	✓	5
*SNOG_10135*	✓	–	✓	✓	–	–	–	–	5
*SNOG_10664*	–	✓	✓	✓	✓	–	✓	–	5
*SNOG_11632*	✓	–	–	✓	✓	✓	–	✓	5
*SNOG_12350*	✓	✓	–	✓	–	✓	–	✓	5
*SNOG_12382*	✓	✓	✓	✓	–	✓	–	–	5
*SNOG_12564*	✓	–	✓	✓	–	✓	–	✓	5
*SNOG_12748*	✓	–	✓	✓	–	✓	–	✓	5
*SNOG_13126*	✓	✓	–	✓	–	✓	–	✓	5
*SNOG_13993*	✓	✓	✓	✓	–	–	–	✓	5
*SNOG_14135*	✓	–	✓	✓	✓	–	–	✓	5
*SNOG_14618*	✓	–	✓	✓	–	–	✓	✓	5
*SNOG_14826*	✓	–	✓	✓	–	✓	–	✓	5
*SNOG_14955*	–	✓	✓	✓	–	✓	✓	–	5
*SNOG_15074*	✓	–	–	✓	✓	✓	–	✓	5
*SNOG_15150*	✓	–	✓	✓	–	✓	–	✓	5
*SNOG_15417*	✓	–	✓	✓	–	–	–	–	5
*SNOG_15989*	✓	–	✓	✓	–	✓	–	✓	5
*SNOG_16091*	✓	–	✓	✓	–	✓	–	✓	5
*SNOG_16131*	✓	–	✓	✓	–	✓	–	✓	5
***SNOG_16166* (scaffold 44)**	–	✓	✓	✓	–	✓	✓	–	5
***SNOG_16226* (scaffold 44)**	✓	✓	–	✓	✓	–	–	✓	5
***SNOG_16236* (scaffold 44)**	✓	✓	–	✓	–	–	✓	✓	5
***SNOG_16237* (scaffold 44)**	✓	✓	–	✓	✓	–	✓	–	5
***SNOG_16270* (scaffold 45)**	–	✓	✓	✓	✓	–	✓	–	5
***SNOG_16345* (scaffold 45)**	–	✓	✓	✓	✓	✓	–	–	5
*SNOG_20011*	✓	✓	–	✓	–	✓	–	✓	5
*SNOG_30026*	✓	–	–	✓	✓	✓	–	✓	5
*SNOG_30316*	✓	–	✓	✓	–	✓	–	✓	5
*SNOG_30334*	✓	–	✓	✓	–	✓	✓	–	5
*SNOG_30645*	✓	–	✓	✓	–	✓	–	✓	5
*SNOG_30701*	✓	–	✓	✓	–	✓	–	✓	5

## Discussion

This comparative analysis of the pan-genomes of *P. nodorum*, *P. avenae*, and *P2* identified dynamic genome regions in the SN15 reference relative to isolates derived from diverse locations and hosts. Analyses of PAV identified several sequences that may be dispensable or lineage specific. Among these, *P. nodorum* SN15 scaffolds 44 and 45 are predicted to collectively encode 136 genes and have distinctive profiles of gene density and positive selection that resemble the “two-speed” genome compartmentalization observed in the genomes of other fungal phytopathogens (Testa et al. 2016), including *L. maculans* (Fudal et al. 2009; Van de Wouw et al. 2010; Rouxel et al. 2011), *Zymoseptoria tritici* (Croll et al. 2015; Goodwin et al. 2011), and *Fusarium oxysporum* (Ma et al. 2010). We speculate that these scaffolds may comprise one or more accessory chromosomes or regions (Covert 1998; Croll and McDonald 2012). Their absence from Sn79 demonstrates that the genes on these scaffolds are not strictly required for survival, and their retention in more virulent strains suggests that they may confer some advantages in some host environments. The strong positive selection observed in SN15 scaffolds 44 and 45 also suggests that the localized accumulation of mutations in these putative accessory regions may promote genetic innovation (Croll and McDonald 2012; Oliver 2012; Croll et al. 2013). Interestingly, 4 of the top 63 effector candidate loci (score ≥ 5, table 5) resided on SN15 scaffold 44 (*SNOG_16166*, *SNOG_16226*, *SNOG_16236*, and *SNOG_16237*) and 2 more (*SNOG_16270* and *SNOG_16345*) resided on SN15 scaffold 45 (supplementary file S1 and table S3, Supplementary Material online). Scaffolds 50 and 55 in isolate WAC8410 also exhibited the low gene density typical of accessory chromosomes (Covert 1998; Goodwin et al. 2011), as well as an extremely high frequency of pseudogenes with identifiable functional paralogs. Collectively, these anomalous scaffolds share many of the features typically associated with accessory chromosomes of other plant pathogens (Goodwin et al. 2011; Croll et al. 2015).

**Fig. 6.**
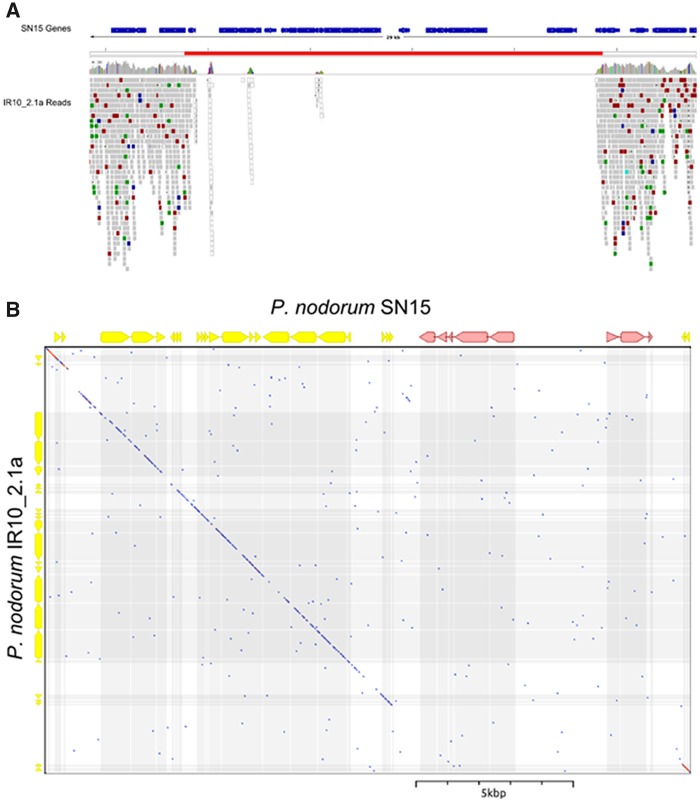
—Presence–absence detection is more accurate using alignments of *de novo* assembled sequences than read mappings. (*A*) Mapped read depth of a region on scaffold_004 in the SN15 reference assembly shows a putative sectional absence of seven genes. (*B*) Dotplot of the alternate strain’s (*P. nodorum* IR10_2.1a) *de novo* assembly at the region (marked red in A) shows that only two of the reference genes (marked in pink) are absent in the alternate strain. Highly variable regions around sectional absences can frustrate mapping algorithms leading to an inflated estimation of absent genes.

By surveying the SNP distribution and density, we identified an oversight associated with previously used short-read sequencing approaches. Previous genome comparisons between *P. nodorum* species used the depth of reads mapped to the reference genome to infer gene absence in the alternate strain (Syme et al. 2013). Regions that are highly differentiated relative to the reference genome can prevent reads from mapping to the reference, inflating the count of genes absent in the alternate strain. Comparisons based on *de novo* assemblies of the alternate strain produced a greater coverage of the reference genome for both *P. nodorum* and *P. avenae*. Figure 6 illustrates this using a region in the reference genome where reads from *P. nodorum* IR10_2.1a failed to map. The region without mapped reads covers 20 kb and includes eight reference genes. A protocol that calculates gene absence from mapping data alone would describe this as an 8-gene sectional absence. However, using nucmer to align *de novo* assembled sequences of this strain to the reference reveals that the absence is only 8 kb and that only two genes are absent from the alternate strain. The surrounding regions are sufficiently variable to prevent the read mapping algorithms from mapping reads and providing coverage. In variable genomic regions that exceed the tolerances of read mapping algorithms, a hybrid mapping/assembly approach to variant detection is likely to provide a more complete picture of sequence differences.

The *Parastagonospora* spp. pan-genome data set made it possible to assess positive selection for almost every locus of the reference isolate SN15. The frequency distribution of genes under diversifying selection allowed us to divide the genome into three categories: (1) a background rate of 5–10% genes under diversifying selection, which represented the majority of the genome; (2) large regions where 20–30% of genes are under diversifying selection. Examples include the putative accessory sequences on scaffolds 44 and 45; and (3) small islands of genes adjacent to repetitive regions under diversifying selection, such as scaffolds 7, 15, and 20 (supplementary tables S3 and S5, and fig. S4, Supplementary Material online).

We hypothesize that these categories may relate to the biological function of the genes they contain. The first category is likely to contain the bulk of the stable genome and the housekeeping genes undergoing long term and continuous adaptation to the environment. The second category represents accessory genomic regions involved in rapid changes such as adaptation to new hosts species or genotypes. Genes contained in regions of the third category may represent coordinately regulated gene clusters such as those involved in secondary metabolism. These category 3 regions are adjacent to repeat-rich stretches of the genome, which in other species are associated with secondary metabolite synthesis gene clusters (Dallery et al. 2017). RIP is known to encroach from repetitive regions into nearby single-copy genes, in some cases disabling recognized effector genes (Fudal et al. 2009; Rouxel et al. 2011). In the case of genes encoding avirulence effectors, mutation can be beneficial to the pathogen by enabling evasion of recognition on a host carrying the corresponding resistance gene. Islands of positive selection were observed to often be adjacent to AT-rich regions (Testa et al. 2016), which suggests there may be an association between RIP and effector loci in *P. nodorum*.

The known *P. nodorum* effector genes are all positioned near repetitive sequences (Syme et al. 2013; Kellner et al. 2014). It has been suggested that repetitive elements provide a mechanism for effector mobilization within genomes and HGT between species, while also increasing diversity at these loci (Croll and McDonald 2012; Oliver 2012; Croll et al. 2013). Notably, a putative *SnTox3* ortholog was observed in both *P. avenae* Pat5 isolates, within a gene-dense region that is otherwise syntenic to the other strains sequenced and shows no sign of nearby transposon activity (fig. 5). The evolutionary history of these homologs at two different loci is unclear. Possible explanations include independent acquisition of the gene by *P. nodorum* and Pat5 ancestors, or lateral transfer between these taxa. Another possibility is that the location in Pat5 is the ancestral version, and subsequent translocation in *P. nodorum* adjacent to a repeat-rich region may provide an adaptive advantage in the form of RIP-mediated accelerated mutation rates (Fudal et al. 2009; Van De Wouw and Howlett 2011; Testa et al. 2016). The absence of any isolate with a copy in both locations likely indicates either direct translocation or transduplication with subsequent loss of the original paralog, likely due to selection pressures imposed by RIP on duplicated sequences (Galagan and Selker 2004; Hane and Oliver 2008).

The addition of new genome assemblies allowed us to expand the set of criteria used to identify candidate effector genes. The presence/absence allele frequency in a *P. nodorum* population differs for each effector, and was hypothesized to reflect the prevalence of each effector’s susceptibility gene in the corresponding host population (McDonald et al. 2013). *SnToxA*, *SnTox1* and *SnTox3* all showed presence/absence polymorphisms, and each were individually observed in 50–85% of the *P. nodorum* isolates studied (fig. 1). A more accurate determination of core and strain-specific genes (fig. 3 and table 3) allows us to better identify genes that are neither perfectly conserved nor infrequent. Based on updated criteria for ranking effector candidate loci, *SnTox1* is among the eight equally top-ranked candidates and *SnTox3* and *SnToxA* are among the nine 2nd-ranked candidates (table 5 and supplementary table S3, Supplementary Material online). The 63 top-scoring candidates will be prioritized for purification in a heterologous expression system and screened against wheat lines to test for each candidate effector’s ability to produce disease symptoms. We anticipate that *P. nodorum* effectors that have not yet been identified to the gene level (SnTox2, SnTox4, SnTox5, and SnTox6) may be able to be matched to these candidates using PAV profiles across the sequenced isolates (e.g., SN4, SN79, and Sn99CH 1A7a) to help unmask these recalcitrant loci. Once validated, effector molecules can be applied as tools to accelerate disease resistance breeding programs (Vleeshouwers and Oliver 2014).

Analyses of the *P. nodorum* and *P. avenae* pan-genomes allowed us to quantify different types of genomic variation across a variety of scales. *De novo* assembly comparisons highlighted the large number of strain-specific loci and the extent of PAV within the two species. Accessory regions were identified, suggesting the possibility of one or more lineage-specific dispensable chromosomes with potential roles in pathogenicity (Zolan 1995; Covert 1998). At the smallest scale of resolution, the pan-genomic comparisons identified loci under diversifying selection. These observations collectively deepen our understanding of the genomic history of *Parastagonospora* spp. and improve the prediction of potential effector sequences in *P. nodorum*. We expect that our approach can be broadly applied to other species in the Pezizomycotina, particularly to those which are necrotrophic pathogens.

## Supplementary Material

Supplementary data are available at *Genome Biology and Evolution* online.

## Supplementary Material

Supplementary Data
