# Supplementary material for: Pan-Parastagonospora Comparative Genome Analysis—Effector Prediction and Genome Evolution
Source: Genome Biol Evol. 2018 Sep 4;10(9):2443–57. doi: 10.1093/gbe/evy192 (PMC6152946; doi:10.1093/gbe/evy192)
Supplement: Supplementary Data [file evy192_supp.zip › S2 Table.docx]

**S2 Table:** Counts of strain-specific proteins for each isolate sequenced.

| Strain | Strain Specific Loci |
| --- | --- |
| A1 3.1a | 178 |
| H6.2b | 132 |
| SN11IR_6_1.1 | 95 |
| SN11IR_7_2.3 | 105 |
| Jansen 4_55 | 624 |
| IR10_5.2b | 63 |
| Hartney99 | 107 |
| SN11IR_2_1.1 | 226 |
| Mt.Baker | 571 |
| s258 | 154 |
| 82-4841 | 101 |
| 83-6011-2 | 102 |
| OH03 Sn-1501 | 15 |
| AR1-1 | 13 |
| VA 5-2 | 15 |
| SNOV92X D1.3 | 19 |
| GA9-1 | 9 |
| MD4-1 | 16 |
| SnOre11-1 | 9 |
| Sn Cp2052 | 54 |
| BRSn9870 | 209 |
| Sn99CH 1A7a | 21 |
| WAC8410 | 71 |
| SWE-3 | 32 |
| FIN-2 | 33 |
| SnChi01 40a | 22 |
| SnSA95.103 | 532 |
| C1.2a | 30 |
| IR10_9.1a | 131 |
| B2.1b | 64 |
| SN79 | 56 |
| SN4 | 252 |
| SN15 | 108 |
