# Supplementary material for: Pan-Parastagonospora Comparative Genome Analysis—Effector Prediction and Genome Evolution
Source: Genome Biol Evol. 2018 Sep 4;10(9):2443–57. doi: 10.1093/gbe/evy192 (PMC6152946; doi:10.1093/gbe/evy192)

**Supplementary figure S3:** By taking a non-overlapping 100kb sliding window over the SN15 reference genome, we calculated the number of genes in the window and the percentage of those genes that are under diversifying selection. Scaffolds 7, 15, 20, 44, and 45 show multiple windows that contain more than 20 genes of which more than 20% are under diversifying selection. Scaffolds 44 and 45 in particular show no windows with less than 15% of genes under diversifying selection.


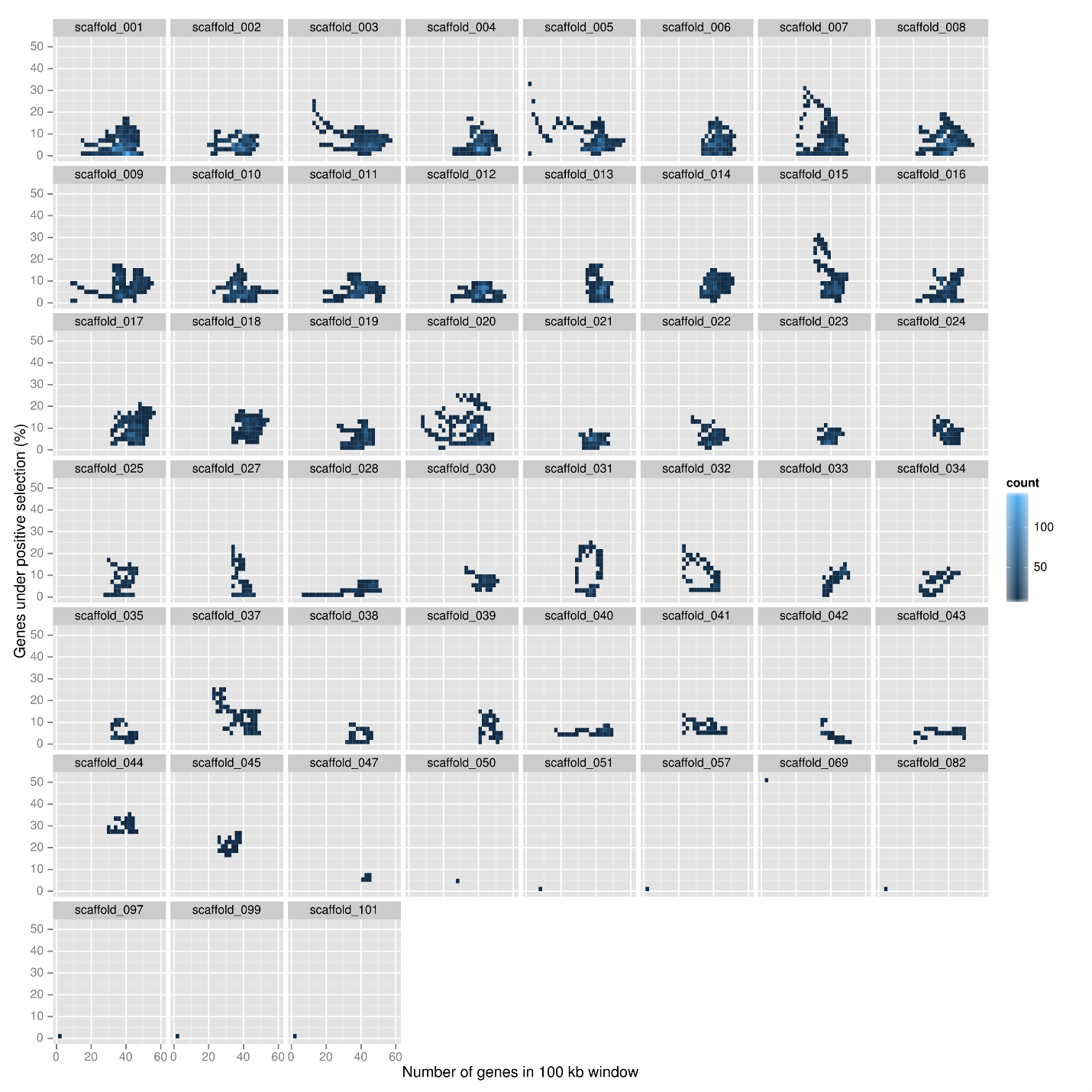

Supplement: Supplementary Data [file evy192_supp.zip › S4 Fig.docx]
