# Supplementary material for: Pan-Parastagonospora Comparative Genome Analysis—Effector Prediction and Genome Evolution
Source: Genome Biol Evol. 2018 Sep 4;10(9):2443–57. doi: 10.1093/gbe/evy192 (PMC6152946; doi:10.1093/gbe/evy192)
Supplement: Supplementary Data [file evy192_supp.zip › Supporting Information Legends.docx]

Supporting Information Legends

S1 File. Gene annotations for all strains in GFF3 format.

S2 Table. Counts of strain-specific proteins for each isolate sequenced.

S3 Table. Summary of effector prediction scores, ranking and supporting evidence.

S4 Fig. Heatmaps of positively selected gene frequency in all SN15 genic scaffolds. By taking a non-overlapping 100kb sliding window over the SN15 reference genome, the number of genes in the window and the percentage of those genes that are under diversifying selection was calculated. Scaffolds 7, 15, 20, 44, and 45 show multiple windows that contain more than 20 genes of which more than 20% are under diversifying selection. Scaffolds 44 and 45 in particular show no windows with less than 15% of genes under diversifying selection.

S5 Table. Summary of significance tests for diversifying selection in SN15 loci compared to alternate strain orthologs.

S6 Table. Predicted ortholog groups and their member genes from all *P. nodorum* and *P. avenae* strains.
